# Novel mutant camelina and jatropha as valuable feedstocks for biodiesel production

**DOI:** 10.1038/s41598-020-78680-w

**Published:** 2020-12-14

**Authors:** Muhammad Mahran Aslam, Asif Ali Khan, Hafiza Masooma Naseer Cheema, Muhammad Asif Hanif, Muhammad Waqar Azeem, Muhammad Abubakkar Azmat

**Affiliations:** 1 Department of Plant Breeding and Genetics, University of Agriculture, Faisalabad, 38040 Pakistan; 2 Muhammad Nawaz Sharif Agriculture University, Multan, Pakistan; 3 Department of Chemistry, University of Agriculture, Faisalabad, 38040 Pakistan; 4 Department of Plant Breeding and Genetics, University of Agriculture, (Burewala Campus), Faisalabad, 38040 Pakistan

**Keywords:** Environmental sciences, Energy science and technology

## Abstract

Novel mutant camelina has become a crop of interest inspired by its short growing season, low harvesting costs and high oil composition. Despite those advantages, limited research has been done on novel mutant lines to determine applicability for biodiesel production. Jatropha is an extremely hardy, frugal and high oil yielding plant species. The major aim of the present study was not only to compare biodiesel production from jatropha and camelina but was also to test the efficacy of camelina mutant lines (M6 progenies) as superior feedstock. The biodiesel yield from camelina oil and jatropha oil was 96% and 92%, respectively. The gas chromatographic analysis using flame ionization detector (GC-FID) showed that mutant camelina oil biodiesel sample contain major amount of oleic acid (46.54 wt%) followed by linolenic acid (20.41 wt%) and linoleic acid (16.55 wt%). Jatropha biodiesel found to contain major amount of oleic acid (45.03 wt%) followed by linoleic acid (25.07 wt%) and palmitic acid (19.31 wt%). The fuel properties of produced biodiesel were found in good agreement with EN14214 and ASTM D6751 standards. The mutant camelina lines biodiesel have shown comparatively better fuel properties than jatropha. It has shown low saponification value (120.87–149.35), high iodine value (130.2–157.9) and better cetane number (48.53–59.35) compared to jatropha biodiesel which have high saponification value (177.39–198.9), low iodine value (109.7–123.1) and lesser cetane number (47.76–51.26). The results of the present student of utilizing novel mutant camelina lines for biodiesel production are quite promising and are helpful in turning out the outcomes of the previous studies suggesting that *C. sativa* biodiesel presents serious drawbacks for biodiesel applications.

## Introduction

Energy is an essential element for all types of economic and social development. Renewable energy technologies use the natural phenomena of converting feedstock into useful forms of energy^1^. The socioeconomic impacts of providing power through renewable resources on a local economy instead of conventional generation technologies are very important. The four most important reasons why the world needs biofuels are (i) Combating climate change (ii) Responding to higher energy consumption (iii) Securing energy supply (iv) Making the most of scarce resources. Combating climate change making to look for alternative energy and fuel sources having low carbon. By the 2050, the world population expected to increase to 8 or even 10.5 billion. In addition, there will be substantially increased energy consumption due to growth in emerging economies. Increasing energy demand in future will pose critical challenges to security of energy supply as fuel resources are scattered around the globe. Reducing the wasted energy amount and making the most of our valuable natural resources is another important crucial thing for our future survival. Biofuels offer a solution to all these problems^2–17^.

Biodiesel is the most valuable form of renewable energy that can be used directly in any existing, unmodified diesel engine. Biodiesel is best renewable substitute for diesel engines^4,18,19^. Chemically, biodiesel is made up of methyl esters of fatty acid obtained by the transesterification reaction of vegetable oil with alcohol^18,20^. Biodiesel burns much cleaner with low emissions of pollutants than petroleum diesel. On combustion, it produces fewer air pollutants such as carbon monoxide, particulates, hydrocarbons, sulfur dioxide, and air toxics^21–23^. Energy Scientists are exploring different cheap biodiesel feedstock to divert the world from high priced pollution producing fossil fuel to the alternate renewable energy resources. The potential of using biodiesel as a fuel for combustion engine has opened up a new horizon for using a wide range of feedstocks for extraction of oil grouped as edible oils, non-edible oils, algal oils (both micro and macroalgae) and genetically engineered plant oils^4^. Microalgae are also being utilized for biodiesel production and its utilization is dependent upon its biomass, fatty acid profile and lipid productivity^24,25^. The genetically engineered feedstocks are most recent feedstock for biodiesel production^26^. There is no doubt that Jatropha is a good feedstock for biodiesel production at mass scale. However, it is a poisonous plant and its crude oil (also called hell oil) is carcinogenic for human skin, the seed is poisonous for human as only 4–5 seeds ingestion is enough to cause death^27^. Its negative impact in case of mono-cropping in flora and fauna of terrestrial and aquatic life has been reported. It has been declared as the natural disaster by the environmentalists^28^. On critical comparison between camelina and jatropha following extremely important fact were found and all these observations supports the use of mutant camelina lines for biodiesel production. As for as the comparison of jatropha with camelina for biofuel production is concerned a detailed study in Thailand (one of the biggest growers of Jatropha) showed that camelina has the net energy ratio as high as 5.22 as compared to the Jatropha with 3.74. *C. sativa* have positive energy balance even for the production of biodiesel only (net energy ratio = 1.47) whereas Jatropha has negative energy balance based on such criterion (net energy ratio = 0.68)^29,30^. One of the myths about jatropha is that it can grow anywhere without care, but studies have shown that in the initial 3–4 years it needs more irrigation water than any other cultivated crops. The cultivation of Jatropha is not economical unless proper inputs are provided^31,32^. Camelina seeds contain average 37% of oil 25–45% protein, 35–49% lipid^32,33^. Camelina oil naturally contains over 50% fatty acids including oleic acid (14.5–19.7%), linolenic acid (32.6–38.2%), linoleic acid (16.9–19.6%) and gadoleic acid (12.4–16.2%). The fatty acid composition of camelina oil varies for different genotypes under a range of locations and environmental conditions^31,32^. These unsaturated fatty acids are good for cardiovascular health and heart because it reduces the low-density lipoprotein (LDL) and cholesterol level in the blood. The oil contains many natural antioxidants, such as tocopherol, which enhance the shelf life of oil^34^. Many previous studies critically noted that camelina is not a good feedstock as its biodiesel presents serious drawbacks for biodiesel applications. These draws backs were found due to exhibited the poorest oxidative stability, highest distillation temperature and has the highest potential to form coke during combustion, all which attributed to the high percentage of polyunsaturated fatty acid (n − 3) methyl esters in camelina oil^35–37^.

The present study reports the comparative use of novel mutant camelina and jatropha seeds oils as a low-cost feedstock for biodiesel-production. The findings of the present study will be helpful in commercializing novel mutant camelina for biodiesel production.

## Material and methods

### Materials

The seed of 3000 camelina mutant lines (M5 progenies) were obtained from the Department of Biological Sciences, University of California Davis, USA. The seeds of these lines were sown under randomized complete block design in three replications to get M6 progenies at the experimental farm of Department of Plant Breeding and Genetics, University of Agriculture, Faisalabad-Pakistan. Fifty high yielding and drought tolerant lines were selected from 3000 mutant lines. The oil was extracted from the seed of harvested 50 mutant M6 drought tolerance lines. Jatropha oil was obtained from the local market of Faisalabad, Pakistan. Methanol, KOH, H_2_SO_4_, Na_2_S_2_O_3_.5H_2_O, and Wijs reagent (Iodine chloride) used in the present study were of analytical grade and purchased from Merck.

### Extraction of oil and production of biodiesel

Healthy seeds of camelina from M6 population obtained by M5 mutant lines were washed, sun dried, and oil was extracted from cold press method. The jatropha oil was purchased from the local market of Faisalabad, Pakistan. Cold pressed seed oils of mutant camelina and jatropha were transesterified into biodiesel and glycerol (by-product). For acid catalyzed transesterification reaction, a mixture of 25 g of mutant camelina/jatropha seed oil, 6:1 methanol to oil molar ratio and required amount of H_2_SO_4_ (25%, 50% and 100% in weight as compared to oil) was heated at 60 °C for 4.5 h with constant stirring. For base catalyzed transesterification, a mixture of 25 g of mutant camelina/jatropha seed oil, 1:3 methanol to oil molar ratio and required amount of KOH (0.125%, 0.25% and 0.5% in weight compared to oil) was heated at 60 °C for 1.5 h with constant stirring. The produced biodiesel was separated from reaction mixture by separatory funnel after the settling of solution in two layers, upper biodiesel layer and the lower glycerol layer. Biodiesel was washed with hot water for the removal of soap and excess methanol.

### Determination of fuel properties

Fatty acid composition of methyl esters was determined by GC-FID analysis. These analyses were performed at University of Agriculture Faisalabad. HI-8014 HANNA instruments pH meter was used for measuring pH of the produced biodiesel. For the determination of density (g/ml) of all biodiesel samples, mass of 1 ml of each produced biodiesel sample was weighed. Standard specific gravity bottle was used to determine specific gravity of biodiesel. The iodine value (IV) (Degree of unsaturation) was measured by dissolving the 0.5 g of biodiesel in 20 ml of CCl_4_ and 25 ml of Wijs solution which taken in glass stoppered iodine flask of 250 ml capacity. The flask was vigorously shaken and placed in the dark for half an hour. Then added 15% solution of KI and 100 ml of distilled water. The solution was titrated against 0.1 N Na_2_S_2_O_3_.5H_2_O using starch as indicator till the disappearance of yellow color of iodine. The procedure was repeated for the blank. The IV was calculated using following formula^18^:

$$IV=\left(S-B\right)\,Normality\,of\, {Na}_{2}\,{S}_{2}{O}_{3}.5{H}_{2}O\times \frac{12}{sample\, wt. (g)}\times 100$$

where S and B represent the volume of titrant used for sample and blank.

For the determination of saponification value (SV) of biodiesel, 0.5 g biodiesel sample and 20 ml alcoholic KOH solution was taken in a 250 ml round bottom flask connected with a condenser. The mixture was heated gently until completion of saponification reaction (a clear solution was indication of completion of reaction). Two drops of phenolphthalein indicator were added into reaction mixture at room temperature. The solution was titrated against 0.5 N HCl until the pink color disappeared. Same procedure was used to measure the bank value. It was measured by using the following formula^18^:

$$Saponification\, value=\frac{(B-S)\times N\times 56.1}{W}$$

where B and S are volume of titrant (ml) used for the blank and biodiesel sample while W is the mass of biodiesel sample used, N is the normality of HCl solution and 56.1 is the molecular weight of KOH (g/mol).

The acid value of biodiesel was measured by taking 0.5 g of biodiesel, 10 ml of ethanol and 1–2 drops of phenolphthalein in a 250 ml titration flask. It was titrated against 0.1 N NaOH solution until the appearance of pink color. Free fatty acids (FFA) were calculated from following formula^38^.

$$\%FFA=\frac{V\times N\times 282}{W}\times 100$$

where V and N is volume and normality of NaOH titrant used, weight of biodiesel used and 282 is the molecular weight of oleic acid as equivalent molecular weight of oil. The percentage of FFA free fatty acid can be converted to acid value by the following formula^18^.

$$Acid \,value=1.989\times \%FFA$$

Cetane number (CN) of the produced biodiesel samples was calculated using iodine value (IV) and saponification value (SV) by the following equation^39^.

$$CN=46.3+\frac{5458}{SV}-0.225\times IV$$

## Results and discussions

### Effect of catalysts on biodiesel yield (%)

**Figure 1:**
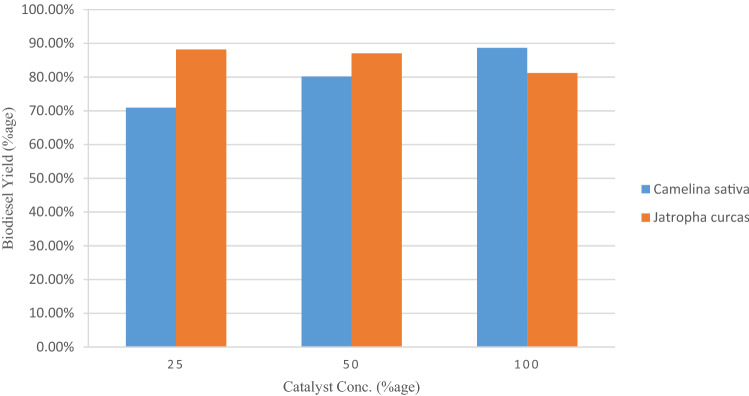
Effect of H_2_SO_4_ concentration on biodiesel yield (%) from Camelina and Jatropha seed oil.

**Figure 2:**
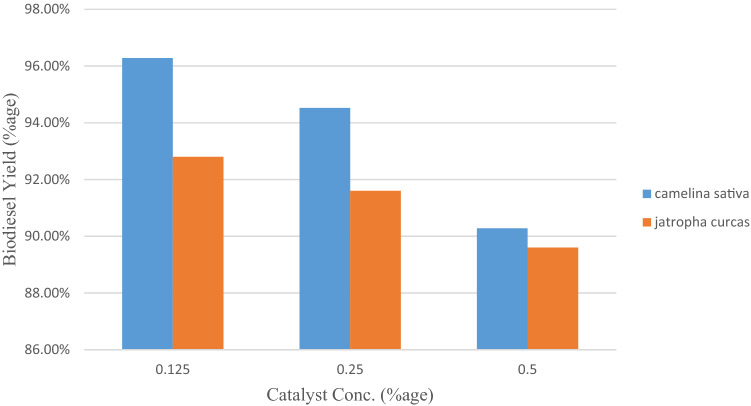
Effect of KOH concentration on biodiesel yield (%) from Camelina and Jatropha seed oil.

Effect of catalyst concentration for maximizing the biodiesel yield from mutant camelina and jatropha seed oil was evaluated by using H_2_SO_4_ and KOH as catalysts at three concentrations shown levels (Figs. 1, 2). Maximum yield of biodiesel was recorded to be 96.28% and 92.8% for mutant camelina and jatropha seed oil at 0.125% KOH as alkaline catalyst with a reaction conditions of 3:1 oil to methanol molar ratio, 60 °C temperature, 1.5 h reaction time at constant stirring (120 rpm). In base catalyzed transesterification reaction higher catalyst concentrations resulted in reduced biodiesel production. Bases favor esterification reaction along with transesterification at higher concentrations which results in the production of soap. This not ultimately reduce biodiesel yield but also makes separation of byproducts from biodiesel difficult. It was reported in a previous study that camelina biodiesel conversion rate decrease by increase the concentration and the maximum biodiesel yield (92.6%) was obtained at conventional homogeneous alkaline-catalyst concentration (0.75 wt.%) at 40 °C for 40 min with 8:1 methanol to oil molar ratio^20^. In case of acid catalyzed transesterification reaction, biodiesel yield increased with increase in the H_2_SO_4_ concentration for mutant camelina oil. A similar trend of biodiesel production was reported in a previous studied biodiesel production from waste tallow^40^. A slight decrease in biodiesel yield was recorded for jatropha oil. The biodiesel produced from mutant camelina was not only better in respect of yield but was also have better stability and fuel properties when compared to camelina biodiesel reported in the previous studies^35–37^. Optimized concentration for biodiesel yield was obtained at 0.125% of KOH catalyst. Mutant camelina (96.28%) exhibited higher biodiesel yield than jatropha biodiesel yield (92.8%). In short, mutant camelina could be another new potential feedstock for biodiesel production along with jatropha.

### Characterization of biodiesel profile

The fuel properties of biodiesel are related to the nature of fatty acids present in the feedstock oil. The degree of unsaturation and chain length determine the physical properties of biodiesel. Tranesterification does not alter fatty acid composition of raw feedstocks^41^. The fatty acid composition of studied biodiesel was shown in Table 1. Triglycerides present in vegetable oil have great potential for biodiesel production, but its quality depends upon the composition of oil. The good quality of biodiesel produced from the oil which have high amount of monounsaturated fatty acid and lower saturated and polyunsaturated fatty acid. The biodiesel produced from sunflower oil have poor oxidation stability due to high polyunsaturated fatty acid. Similarly biodiesel production from palm oil have poor flow properties or may be solid at room temperature and perform better in temperate regions^42^. The mutant camelina and jatropha biodiesel profile was identified by gas chromatographic analysis equipped with flame ionization detector (GC-FID).

**Table1:** The fatty acid composition of mutant camelina and jatropha oils.

Fatty acid	C: D	%age contents	
		*Camelina sativa*	Jatropha
Palmitic acid	C16:0	7.34	19.31
Palmitoleic acid	C16:1	1.03	0.63
Stearic acid	C18:0	3.73	4.24
Oleic acid	C18:1	46.54	45.03
Linoleic acid	C18:2	16.55	25.07
Linolenic acid	C18:3	20.41	5.39
Erucic acid	C22:1	4.01	nd

The gas chromatographic analysis using flame ionization detector (GC-FID) (Table 1) showed that mutant camelina oil biodiesel sample contain major amount of oleic acid (46.54 wt%) followed by linolenic acid (20.41 wt%) and linoleic acid (16.55 wt%). Jatropha biodiesel found to contain major amount of oleic acid (45.03 wt%) followed by linoleic acid (25.07 wt%) and palmitic acid (19.31 wt%). The fatty acid composition of mutant camelina was quite variable from camelina oil reviewed previously^43^. Mutant camelina lines found to have higher amounts of oleic acid than non-mutant varieties and could serve as a potential feedstock in biofuel sector in upcoming years. However, jatropha fatty acid profile was similar to previously reported work^44^. One pervious study has reported that biodiesel containing fatty acids more than 15 carbon atoms is considered to be of superior quality^45^. Both mutant camelina and jatropha biodiesels contains high quantity of fatty acids having more than 15 carbon atoms and could serve as good sources for renewable energy.

### Assessment of fuel properties

#### Physical properties

The physical properties of both mutant camelina and jatropha biodiesel were evaluated and summarized in Tables 2, 3, 4and 5. The pH of biodiesel produced from mutant camelina (M6) and jatropha are presented in Table 2. The densities of various jatropha and mutant camelina biodiesel samples were found ranging from 0.67–0.72 g/ml and 0.74–0.89 g/ml, respectively. Most of the values of densities for mutant camelina biodiesel are in the specified range by European standards (0.86–0.90 g/ml) while those of jatropha biodiesel values found below the recommended limits. The maximum density 0.89 g/ml was observed for the biodiesel produced from mutant camelina at 0.50% KOH (w/w% of oil). The minimum value of density (0.68 g/ml) was observed for jatropha biodiesel at catalyst concentration of 100% H_2_SO_4_ (w/w% of oil) (Table 3). The density of biodiesel has influence the spray properties, injection timing and injection system^46^. Greater density of biodiesel, more mass of fuel will be injected in the cylinder which increases the energy of the engine and vice versa. Generally the density of biodiesel decrease with molecular weight and increase with unsaturation level of oil^42^. Viscosity defined as “the resistance in flowing liquid”. The viscosity of mutant camelina and jatropha biodiesel produced by using various acid and alkali catalysts concentrations was 113–119 centipoise (CP) and 64–82 CP, respectively (Table 4). In the previous study 130 centipoise viscosity was recorded in camelina sativa biodiesel^47^. The viscosity of biodiesel decreases with increasing unsaturation level and temperature but increases with molecular weight^48–50^. “The point at which crystals of biodiesel starts to appear known as cloud point”. “The temperature at which fuel converted to gel like appearance which do not flow known as fuel's pour point”. Cloud point of mutant camelina was found varying from − 2 to − 2.5 °C and jatropha from 0.2 to − 2 °C. A lower cloud point makes biodiesel even suitable during winters or in cold areas^47,51^. There is no limit defined for cloud point and pour point in European and American standards and it changes with respect to climatic zone^37^. Higher cloud point causes poor flow problem in the combustion chamber, delayed startup and misfire. The cloud point of biodiesel decreases with increase in the degree of unsaturation and increases by increase in the fatty acid chain length^47,52^. Mutant camelina biodiesel have present good fuel properties for its utilization as renewable energy resource.

**Table 2:** pH of biodiesel produced from mutant camelina and jatropha.

Catalyst	Catalyst conc. (%)	pH	
		Mutant camelina	Jatropha
KOH	0.125	6.82 ± 0.08	7.30 ± 0.03
	0.25	7.62 ± 0.07	7.35 ± 0.04
	0.50	7.98 ± 0.03	8.00 ± 0.03
H_2_SO_4_	25	5.60 ± 0.05	6.38 ± 0.02
	50	5.10 ± 0.02	6.30 ± 0.07
	100	4.67 ± 0.01	5.98 ± 0.01

**Table 3:** Densities (g/ml) of biodiesel produced from mutant camelina and jatropha.

Catalyst	Catalyst conc. (%)	Densities (g/ml)	
		Mutant camelina	Jatropha
KOH	0.125	0.82	0.73
	0.25	0.86	0.70
	0.50	0.89	0.67
H_2_SO_4_	25	0.87	0.72
	50	0.79	0.72
	100	0.74	0.68

**Table 4:** Viscosity (centipoise) of biodiesel from mutant camelina and jatropha.

Catalyst	Catalyst conc. (%)	Viscosity	
		Mutant camelina	Jatropha
KOH	0.125	114 ± 2.86	82 ± 3.71
	0.25	121 ± 2.86	75 ± 1.56
	0.50	118 ± 2.86	68 ± 4.71
H_2_SO_4_	25	119 ± 2.44	76 ± 3.92
	50	116 ± 2.44	64 ± 2.51
	100	113 ± 2.44	69 ± 3.60

**Table 5:** Free Fatty acids in biodiesel produced from mutant camelina and jatropha.

Catalyst	Catalyst conc. (%)	%FFA	
		Mutant camelina	Jatropha
KOH	0.125	0.35 ± 0.03	11.84 ± 1.34
	0.25	0.30 ± 0.73	10.56 ± 1.45
	0.50	0.38 ± 0.21	12.78 ± 0.11
H_2_SO_4_	25	0.43 ± 1.65	12.98 ± 0.55
	50	0.42 ± 2.03	11.32 ± 0.61
	100	0.35 ± 1.23	11.67 ± 0.77

#### Chemical properties

Experimental results showed that mutant camelina and jatropha biodiesel produced by acid and base catalysts have free fatty acid (FFA) in the range of 0.35–0.43% and 10.56–12.98%, respectively (Table 5). FFA and moisture content has significant effect on transesterification of triglycerides with alcohol using catalyst^53^. The high FFA content (> 1% w/w) will produce the soap in large quantities and cause difficulty during the separation of biodiesel layer. This ultimately decrease biodiesel yield^54^. In the present study, the mutant camelina biodiesel was found to contain much lower amount of FFA which is one of requirements for the production of good quality biodiesel^55^. The acid value determined for mutant camelina and jatropha biodiesel were 0.59 to 0.67 and 21 to 23.55, respectively (Table 6). Acid value indicates the concentration of free fatty acids in the biodiesel sample. Experimental results showed that jatropha biodiesel have high free fatty acids as compared to mutant camelina. Jatropha biodiesel exhibited much higher acid values than European and American standards. The saponification values of mutant camelina and jatropha biodiesel produced by acid and base catalysts are summarized in Table 7. The observed saponification values mutant camelina and jatropha were 120.87–149.35 and 177.39–198.9, respectively. The saponification value of 242 was recorded for date palm biodiesel^18^ and 193.33 for jatropha biodiesel previously^53^. Mutant camelina has exhibited quite suitable saponification values for good quality biodiesel production. The iodine value is a measure of the degree of unsaturation of fats and oils. Higher iodine value indicates higher unsaturation in fats and oils^56,57^. The iodine value of mutant camelina and jatropha oil was found to be 130.2–157.9 and 109.7–123.1, respectively (Table 8). Iodine value relies on the unsaturated fatty acid and indicate double bound present in biodiesel sample^58^. According to European standards iodine values equal or less than 120 is preferable for biodiesel produced for consumption. However, in American standards no iodine value is specified. Unsaturation represented by iodine value reflects biofuel chance of solidification^45^. Two previous studies reported camelina biodiesel having iodine values of 151^47^ and 153^52^. Iodine value for mutant camelina biodiesel was observed to be 120 by using catalyst 0.125% KOH at 60 °C for 1.5 h in the present study. Higher iodine values indicate the presence of the higher amounts of unsaturated fatty acids in the biodiesel. Such higher amounts undergo polymerization reactions due to high availability of unsaturated bonds at high temperature in the internal combustion engine. The ultimate result is the deposition or deterioration of biodiesel in the engine^41^. Fuels that have such properties (e.g. safflower oil, sunflower oil and soybean oil) also considered to produce thick sludges in the sump of the engine during fuel seeping down the sides of the cylinder into crankcase^42^. Jatropha oil is placed in the semi-drying oil group according to its iodine values. High iodine values of mutant camelina and jatropha oils are due to the high contents of unsaturation fatty acids such as linoleic acid and oleic acid. Cetane number (CN) is very important quality parameter for evaluating the biodiesel. Generally, it is similar to the octane number of gasoline, and give us information about fuel ignition performance inside the engine^56^. It give idea that how quickly fuel burn during the combustion inside the cylinder of engine. The biodiesel which have higher cetane number ignite quickly and completely. On the contrary, low cetane number lead to incomplete combustion, emission of NOx and may cause knocking during combustion^59^. According to European and American standards, the minimum cetane number is 47 and 51, respectively. The cetane number of conventional petro-diesel is 40 which is lower than biodiesel^56^. It mainly depends on the fatty acid chain length and degree of unsaturation. Cetane number increases by increasing chain length and decreases by increasing number of double bonds^60^. In the present study, the cetane number of mutant camelina biodiesel ranged between 48.53–59.35 and that of jatropha biodiesel ranged between 47.76–51.26 (Table 9). The cetane number of mutant camelina is higher than jatropha and meet well the European and American standards. The difference in cetane number of the biodiesel from mutant camelina and previously reported non mutant camelina is due to slight change in fatty acid profile^52^.

**Table 6:** Acid value of biodiesel produced from mutant camelina and jatropha.

Catalyst	Catalyst conc. (%)	Acid value	
		Mutant camelina	Jatropha
KOH	0.125	0.67 ± 0.07	23.55 ± 1.80
	0.25	0.59 ± 0.07	21 ± 1.80
	0.50	0.75 ± 0.07	25.41 ± 1.80
H_2_SO_4_	25	0.85 ± 0.08	25.81 ± 1.23
	50	0.83 ± 0.08	22.51 ± 1.44
	100	0.67 ± 0.08	23.21 ± 2.80

**Table 7:** Saponification values (mg KOH/1 g) of biodiesel from mutant camelina and jatropha.

Catalyst	Catalyst conc. (%)	Saponification value	
		Mutant camelina	Jatropha
KOH	0.125	137.45 ± 6.37	198.9 ± 2.67
	0.25	129.94 ± 6.98	188.20 ± 2.67
	0.50	149.25 ± 5.43	177.39 ± 2.67
H_2_SO_4_	25	136.5 ± 2.67	191.46 ± 1.32
	50	120.87 ± 5.43	182.71 ± 1.32
	100	121.49 ± 1.32	179.50 ± 1.32

**Table 8:** Iodine values of biodiesel from mutant camelina and jatropha.

Catalyst	Catalyst conc. (%)	Iodine value	
		Mutant camelina	Jatropha
KOH	0.125	120.2 ± 3.67	109.7 ± 2.45
	0.25	142.7 ± 2.98	115.3 ± 1.56
	0.50	152.6 ± 3.78	114.6 ± 1.23
H_2_SO_4_	25	157.9 ± 3.94	120.2 ± 2.01
	50	142.7 ± 2.92	119.7 ± 2.45
	100	153.1 ± 1.45	123.1 ± 2.12

**Table 9:** Cetane numbers of biodiesel from mutant camelina and jatropha seed biodiesel.

Catalyst	Catalyst conc. (%)	Cetane number	
		Mutant camelina	Jatropha
KOH	0.125	56.71 ± 3.55	49.02 ± 3.01
	0.25	56.19 ± 2.87	49.35 ± 4.32
	0.50	48.53 ± 3.96	51.26 ± 2.01
H_2_SO_4_	25	50.75 ± 1.96	47.76 ± 2.92
	50	59.35 ± 4.12	49.23 ± 1.80
	100	56.77 ± 3.68	49.07 ± 2.40

## Conclusions

The research in this paper depicted the feasibility of biodiesel production from mutant camelina seed oil. Mutant camelina seed oil is perhaps the best source of oil for biodiesel production when depletion of petroleum and environmental issues are the major threats in the current century. The fuel properties of both (mutant camelina and jatropha biodiesel) were evaluated and all parameters compared with American and European standards. The mutant camelina biodiesel have high cetane number and low viscosity as compared to jatropha biodiesel. The mutant camelina oil is easily and readily converted into biodiesel as compared to jatropha seed oil and gave 96.28% yield on alkaline transesterification under optimized condition.
